# Inhibition of cystathionine *β*-synthetase suppresses sodium channel activities of dorsal root ganglion neurons of rats with lumbar disc herniation

**DOI:** 10.1038/srep38188

**Published:** 2016-12-01

**Authors:** Jun Yan, Shufen Hu, Kang Zou, Min Xu, Qianliang Wang, Xiuhua Miao, Shan Ping Yu, Guang-Yin Xu

**Affiliations:** 1 Department of Orthopedics, the Second Affiliated Hospital of Soochow University, Suzhou 215004, China; 2 Jiangsu Key Laboratory of Translational Research and Therapy for Neuro-Psycho-Diseases and Institute of Neuroscience, Soochow University, Suzhou 215123, China; 3 Center for translational Medicine, the Affiliated Zhangjiagang Hospital of Soochow University, Zhangjiagang 215600, China; 4 Department of Anesthesiology, Emory University School of Medicine, Atlanta, GA 30322, USA

## Abstract

The pathogenesis of pain in lumbar disc herniation (LDH) remains poorly understood. We have recently demonstrated that voltage-gated sodium channels (VGSCs) in dorsal root ganglion (DRG) neurons were sensitized in a rat model of LDH. However, the detailed molecular mechanism for sensitization of VGSCs remains largely unknown. This study was designed to examine roles of the endogenous hydrogen sulfide synthesizing enzyme cystathionine *β*-synthetase (CBS) in sensitization of VGSCs in a previously validated rat model of LDH. Here we showed that inhibition of CBS activity by *O*-(Carboxymethyl) hydroxylamine hemihydrochloride (AOAA) significantly attenuated pain hypersensitivity in LDH rats. Administration of AOAA also reduced neuronal hyperexcitability, suppressed the sodium current density, and right-shifted the V_1/2_ of the inactivation curve, of hindpaw innervating DRG neurons, which is retrogradely labeled by DiI. *In vitro* incubation of AOAA did not alter the excitability of acutely isolated DRG neurons. Furthermore, CBS was colocalized with Na_V_1.7 and Na_V_1.8 in hindpaw-innervating DRG neurons. Treatment of AOAA markedly suppressed expression of Na_V_1.7 and Na_V_1.8 in DRGs of LDH rats. These data suggest that targeting the CBS-H_2_S signaling at the DRG level might represent a novel therapeutic strategy for chronic pain relief in patients with LDH.

Lumbar disc herniation (LDH) remains a very common and challenging disorder for clinicians. It is defined by recurrent symptoms of low back pain and sciatica. The pathophysiology of pain in LDH involves mechanical compression and chemical inflammation of the nerve roots^1^,^2^. However, the exact causes of low back pain and sciatica have not been fully elucidated and effective therapeutics for the primary symptoms has been unavailable. Recent studies in rodents found that autologous nucleus pulposus (NP) transplantation induced rats to develop pain hypersensitivity^3^,^4^. Therefore, autologous NP transplantation in rats has been used as an animal model of LDH to study the mechanisms of chronic pain.

Evidence showed that LDH involves an increase in excitability of primary afferent nociceptors of dorsal root ganglion (DRG), which convey peripheral stimuli into action potentials (APs) that propagate to the central nervous system. Sensitization of primary sensory neurons is maintained by a number of ion channels such as transient receptor potential channels^5^, purinergic P2X3 receptors^4^, and voltage-gated sodium, potassium and calcium channels^6^,^7^,^8^. VGSCs are integral membrane glycol-proteins that are essential for AP generation and conduction of in excitable cells, thus playing a crucial role in regulating neuronal excitability. Increase in VGSC function and expression may contribute to the enhanced neuronal excitability^9^. The subunits of mammalian VGSCs have been classified into nine different subtypes (Na_V_1.1–Na_V_1.9). VGSCs have been categorized according to their sensitivity to the blocker tetrodotoxin (TTX) wherein the currents carried by Na_V_1.1–1.4, 1.6, and 1.7 are completely blocked, whereas the currents mediated by Na_V_1.5, Na_V_1.8, and Na_V_1.9 are resistant or insensitive to TTX. DRG neurons predominantly express Na_V_1.7, Na_V_1.8 and Na_V_1.9^10^. We have previously showed that VGSCs in DRG neurons were sensitized in this setting^11^. However, the detailed mechanism underlying the sensitization of VGSCs remains unknown.

Recently, we have reported that H_2_S could enhance the sodium current density of DRG neurons from healthy rats^6^,^9^. Therefore, we hypothesize that upregulation of the endogenous H_2_S production enzyme cystathionine *β*-synthetase (CBS) expression sensitizes VGSCs in DRG neurons of rats with autologous NP transplantation, thus leading to pain hypersensitivity. In the present study, we focused on roles of CBS-H_2_S signaling in modulating activities of Na_V_1.7 and Na_V_1.8 of DRG neurons innervating the hindpaw. We showed that administration of CBS inhibitor AOAA significantly reduced neuronal excitability, sodium current density and expression of Na_V_1.7 and Na_V_1.8 of DRGs in LDH rats. Our findings implicate an important role for CBS-H_2_S signaling in modulation of sodium channel activities in a rat model of LDH and identify CBS as a potential molecular target for the treatment of chronic pain in patients with LDH.

## Materials and Methods

### Establishment of LDH model

Surgical procedures for the LDH model were carried out on adult male rats under deep anesthesia, which was induced by intraperitoneal (i.p.) injection of sodium pentobarbital at dose of 50 mg/kg body weight, as described previously^4^. Briefly, NP was harvested from the disc level between the second and the third coccygeal intervertebral disc of rat tail. For implantation of NP, a midline dorsal incision of the skin from L4 to S1 was done over the lumbar spine to expose lumbar 5 and 6 nerve roots. Harvested NP (~5 mg) was then placed on the top of the left L5 and L6 nerve roots close to the corresponding DRGs. Special attention was taken to reduce the mechanical compression and inflammation. Rats in sham group were underwent the same surgical procedures identical to the NP-treated rats but without implantation of the autologous NP. After surgery, animals were returned to individual cage for surgical recovery. Drinking water and food pellets are give free access in the animal research center room. Handling and care of these rats were approved by the Institutional Animal Care and Use Committee of Soochow University. All methods were performed in accordance with the relevant guidelines and regulations of the International Association for the Study of Pain.

### Retrogradely labeling of DRG neurons innervating the hindpaw

DRG neurons innervating the hindpaw were labeled by injection of 1, 1′-dioleyl-3, 3, 3′, 3-tetramethylindocarbocyanine methanesulfonate (DiI, Invitrogen) into the rat left hindpaw, as described previously^4^,^12^. In short, rats were anesthetized with a cocktail of sodium pentobarbital (50 mg/kg body weight, ip). DiI was injected into the plantar skin of left hindpaw (2 μl/site, 4–6 sites). To prevent leakage of the DiI, needle was left in place for 1 min for each injection. Seven days later, left L_5_-L_6_ DRGs were dissected out for immunofluorescence studies or for patch clamp recordings.

### Measurement of pain behaviors

Mechanical and thermal sensitivity of the plantar surface of the hindpaws of NP-rats treated with NS (n =  7) or AOAA (n =  7) were examined 1 day before administration of AOAA and 0.5, 1, 4, 8, 12, 24 and 48 hours after AOAA treatment by an investigator in a blinded manner. Rat hindpaw withdrawal threshold (PWT) in response to stimulation of von Frey filaments was determined as described previously^4^. To measure thermal hyperalgesia, rats were housed in a Plexiglas box on top of a glass platform, and the withdrawal latency (PWL) from a thermal stimulus was obtained by using the radiant heat test with a Model 336 Paw/Tail Stimulator Analgesia Meter (IITC/life Science Instruments, CA, USA), which was set at 2% idle light intensity and 50% working light intensity. The stimulus was turned off manually on the hindpaw withdrawal or automatically if the 20 seconds cut-off time was reached as described previously^13^. Each rat received five trials, 30 seconds apart, with 10 minutes between trials. Results were averaged for analysis. Data were expressed as the latency to withdrawal in seconds.

### Electrophysiological recordings of acutely dissociated DRG neurons labeled with DiI

Seven days after DiI injection, NP-rats treated with NS or AOAA were sacrificed by cervical dislocation, followed by decapitation. Dissected DRGs (Left L_5_-L_6_) were moved to an ice-cold, oxygenated dissecting solution. The dissecting solution included (in mM) 130 NaCl, 5 KCl, 2 KH_2_PO_4_, 6 MgSO_4_, 1.5 CaCl_2_, 10 glucose and 10 HEPES with pH = 7.2 and osmolarity = 305 mOsm. After removal of the connective tissue, these two ganglia were incubated in a 5 ml the solution containing trypsin (~1.5 mg/ml; Sigma, St Louis, Missouri, USA) and collagenase D (~1.8 mg/ml; Roche, Indianapolis, Indiana, USA) for 1.5 hours at 34.5 °C. After digestion, DRGs were taken from the enzyme solution, washed and moved to 0.5 ml of the solution containing DNase (0.5 mg/ml; Sigma, St Louis, Missouri, USA). A single cell suspension was then harvested by repeated trituration via flame-polished glass pipettes. The normal external solution contained (in mM): 130 NaCl, 5 KCl, 2 KH_2_PO_4_, 1 MgCl_2_, 2.5 CaCl_2_, 10 glucose and 10 HEPES with pH adjusted to 7.2 with NaOH, osmolarity adjusted to 295–300 mOsm. The pipette solution was composed of (in mM): 140 potassium gluconate, 10 NaCl, 5 EGTA and 1 CaCl_2_, 10 glucose, 10 HEPES, pH = 7.25 adjusted with KOH; osmolarity = 292 mOsm. The resting potential (RP) and action potentials (APs) of DiI labeled DRG neurons were obtained by an EPC10 patch clamp amplifier (HEKA; Germany) under current clamp conditions at room temperature (RT) around 22 °C. In addition, the numbers of cells with rebound APs was observed. The rebound APs was the APs that recorded after hyperpolarization of cell membrane. Patch clamp data were stored on a personal computer for offline analysis by FitMaster (HEKA; Germany).

### Recording the voltage-gated sodium channel currents

To record the voltage-gated sodium currents, we used the previously developed procedures^14^. In short, the acutely dissociated DRG neurons were superfused (2 ml/min) at RT with an external solution containing (in mM): 60 NaCl, 80 Choline chloride, 0.1 CaCl_2_, 0.1 CdCl_2_, 10 tetraethylammonium-Cl, 10 glucose, 10 HEPES. The pH of the external solution was 7.4 adjusted with tetraethylammonium-OH. The osmolarity was ~300 mOsm adjusted with sucrose. The patch pipette solution contains (in mM): 140 CsF, 1 MgCl_2_, 3 Na-GTP, 5 EGTA, 10 glucose, 10 HEPES, pH = 7.2 adjusted with CsOH, osmolarity = 285~295 mOsm. The total Na_V_ currents of DiI labeled DRG neurons were examined in response to depolarization steps to different testing potentials from −70 mV to +50 mV in 10 mV increments with a pulse duration of 80 ms. The recorded currents were filtered at 2 kHz or 5 kHz and digitally sampled at 50 or 100 μs/point. The current density (pA/pF) was measured by dividing the peak current amplitude by whole cell membrane capacitance, which was obtained by reading the value for whole cell input capacitance cancellation directly from the patch-clamp amplifier. The reversal potentials of voltage-gated sodium channel currents were determined directly or by extrapolation if necessary.

### Immunofluorescence experiments

Seven days after DiI injection, rats were deeply anesthetized and perfused transcardially with 150 mL phosphate-buffered saline (PBS) followed by 400 mL ice-cold 4% paraformaldehyde (PFA) in PBS. Rats were euthanized and the L_5_ and L_6_ DRGs were postfixed in PFA for 1 hour and cryoprotected overnight in 20% sucrose in PBS. As described previously^7^, 10 μm sections of DRG were simultaneously incubated with Na_V_1.7 or Na_V_1.8 (1:200, Alomone lab, Israel) and CBS (1:200, Abnova, CA) antibody. The second antibodies used in the present study were Alexa Fluor 355 and 488. Negative control was determined by omitting the primary antibody. Images of all target proteins were obtained and analyzed by use of Metaview software.

### Western blot analyses

The L_5_ and L_6_ DRGs from LDH rats treated with AOAA or equal volume of normal saline (NS) were harvested and lyzed in 100 μl of radioimmunoprecipitation assay buffer containing 1% NP-40, 0.5% Na deoxycholate, 0.1% SDS, PMSF (10 μl/ml) and aprotinin (30 μl/ml; Sigma, St Louis, Missouri, USA). As described previously, twenty micrograms (20 μg) of proteins were loaded onto a 10% Tris-HCl SDS-PAGE gel (Bio-Rad, Hercules, CA) for detecting Na_V_1.3, Na_V_1.7, Na_V_1.8, Kv1.1 and Kv1.4 expression. After electrically transferring onto polyvinyldifluoride membranes, the immunoreactive proteins were exhibited by enhanced chemiluminescence (ECL kit; Habersham Biosciences, Arlington Heights, IL). The targeted protein bands were determined by exposure of the membrane onto an x-ray film. For quantification of Na_V_1.3, Na_V_1.7, Na_V_1.8, Kv1.1 and Kv1.4 protein levels, photos were digitalized and analyzed by utilizing a scanner (Bio-Rad imaging system). GAPDH was used as a loading control. All protein samples were normalized to GAPDH.

### Application of AOAA

The CBS inhibitor O-(Carboxymethyl) hydroxylamine hemihydrochloride (AOAA) was obtained from Sigma-Aldrich (USA). Immediately after resolved in NS, AOAA was injected intrathecally at 10 μg/kg body weight, once daily for 7 consecutive days. Same volume of NS was used as control. L_5-6_ DRGs from LDH rats after AOAA treatment were collected either for measurement of Na_V_1.7 and Na_V_1.8 expression or for patch clamping studies. For *in vitro* experiment, AOAA at 1 μM was incubated with acutely dissociated DRG neurons for one hour.

### Data analyses

Data are shown as means ± SEM. Normality of all data was examined before analysis. Depending on the data distribution properties, two sample t-test or Dunn’s post hoc test following Friedman ANOVA or Mann-Whitney test or Tukey post hoc test following Kruskal-Wallis ANOVA were used to determine the statistical significance. A value of p < 0.05 was considered statistically significant.

## Results

### CBS inhibitor AOAA treatment attenuates mechanical and thermal hypersensitivity

**Figure 1:**
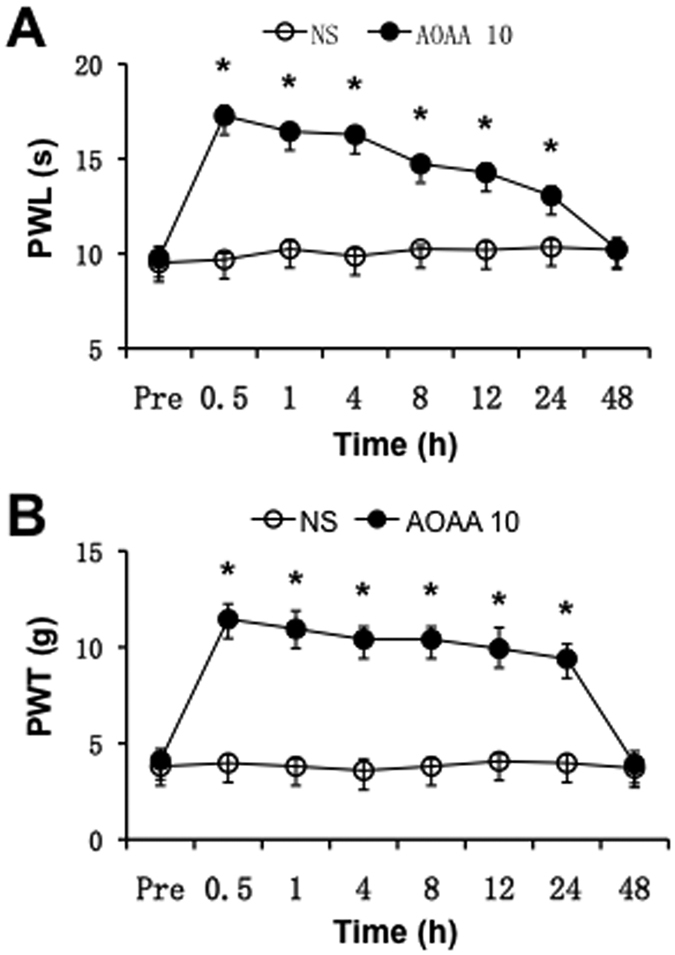
Inhibition of CBS by AOAA attenuated NP-induced mechanical and thermal hypersensitivity. AOAA at 10 μg/kg body weight was intrathecally injected once per day for consecutive 7 days. (**A**) There was significant effect of AOAA on pain withdrawal latency (PWL) to thermal stimulation 30 min after intrathecal injection. The antinociceptive effect returned to baseline level 48 hours after injection (n = 7 rats for each group, *p < 0.01). (**B**) There was significant effect of AOAA on pain withdrawal threshold (PWT) to von Frey filament 30 min after intrathecal injection when compared with NS group. The antinociceptive effect returned to baseline 48 hours after injection of AOAA (n = 7 rats for each group, *p < 0.01).

Sixteen LDH rats were intrathecally injected with AOAA in a volume of 10 μl (10 μg/kg body weight) once per day for consecutive 7 days. As shown in Fig. 1, administration of AOAA significantly enhanced the PWL (Fig. 1A, n = 7 for each group, *p < 0.01) 30 minutes after injection. The antinociceptive effects returned to baseline level 48 hours after last injection of AOAA. In a line with our previously published data^4^, we showed that intrathecal injection of AOAA in a volume of 10 μl markedly enhanced PWT (Fig. 1B, n = 7 for each group, *p < 0.01). There was no significant effect of NS injection on PWT and PWL of LDH rats (Fig. 1A and B, n = 8 rats for each group).

### CBS inhibitor AOAA reverses the enhanced neuronal excitability

**Figure 2:**
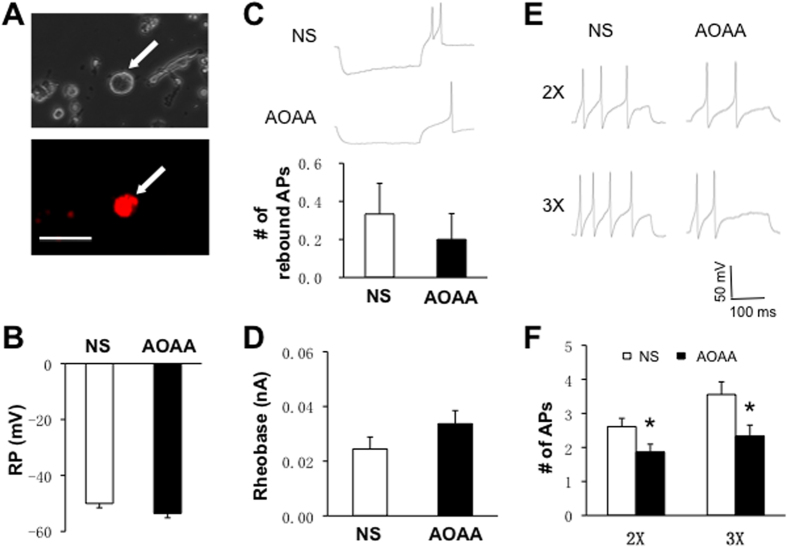
Inhibition of CBS by AOAA reduced neuronal hyperexcitability. (**A**) Representative DRG neuron retrogradely labeled by 1,1′-dioleyl-3,3,3′,3-tetramethylindocarbocyanine methanesulfonate (DiI) viewed under fluorescence microscope (bottom) and bright field (top). Scale bar = 50 μm. (**B**) Compared with the NS group, there was no significant change in resting membrane potential (RP, p > 0.05). (**C**) There was no significant difference of numbers of rebound action potentials (APs) between NS and AOAA group (p > 0.05). (**D**) Compared with the NS group, there was no significant change in rheobase (p > 0.05). (**E**) The representative traces of action potentials (APs) induced by 300-ms depolarizing current injection at 2× rheobase (top) and 3× rheobase (bottom) in L_5–6_ DRG neurons from NS (left) and AOAA group (right) under current-clamp conditions. (**F**) AOAA treatment (intrathecally once per day for consecutive 7 days after NP-transplant) resulted in a significant decrease in the number of APs induced by a 2× and 3× rheobase current injection in L_5–6_ DRG neurons (*p < 0.05).

To determine whether AOAA treatment reverses hyperexcitability of L_5_-L_6_ DRG neurons of LDH rats, we measured cell membrane properties including resting membrane potential (RP), rheobase and the numbers of action potentials (APs) evoked by rheobase current stimulation of DiI-labeled DRG neurons (Fig. 2, arrow, bottom). DRG neurons innervating the hindpaw were labeled by DiI (Fig. 2A, arrow, bottom). Compared with the NS-treated group, there was no significant change in RPs (Fig. 2B), the number of rebound APs (Fig. 2C) and rheobase (Fig. 2D) in AOAA-treated group. However, AOAA treatment significantly reduced the numbers of APs in responding to 2 times and 3 times rheobase current stimulation (*p < 0.05, Fig. 2E and F). The numbers of AP evoked by 2× rheobase current stimulation were 2.6 ± 0.2 (n = 18 cells) and 1.9 ± 0.2 (n = 16 cells) from NS and AOAA-treated rats, respectively. The numbers of AP evoked by 3× rheobase current stimulation were 3.6 ± 0.4 (n = 18 cells) and 2.3 ± 0.3 (n = 16 cells) for NS and AOAA-treated rats, respectively.

**Figure 3:**
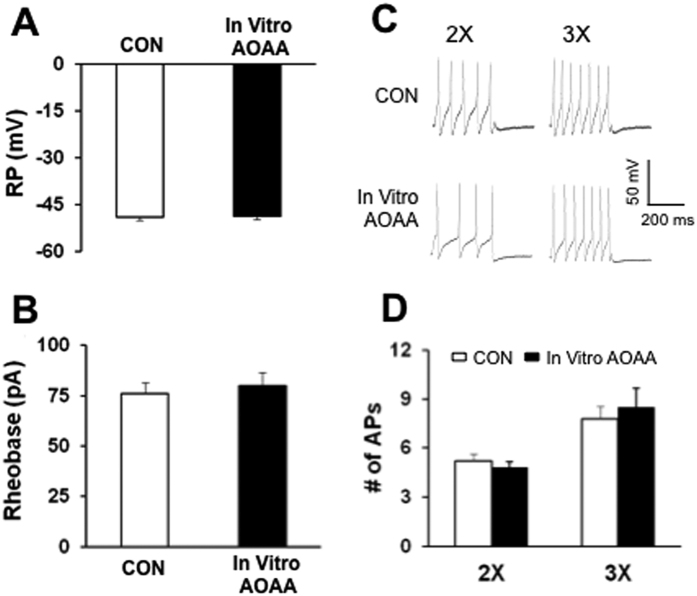
No effect of AOAA incubation *in vitro* on neuronal excitability. (**A**) Resting membrane potential, (**B**) Rheobase, (**C**) and (**D**) numbers of action potentials evoked by 2 times and 3 times rheobase current stimulation were not altered after *in vitro* incubation of AOAA at 1 μM for one hour.

We next determined the effect of AOAA *in vitro* incubation on neuronal excitability. As shown in Fig. 3, *in vitro* application of AOAA at 1 μM for one hour did not significantly alter the resting membrane potentials (Fig. 3A), rheobase (Fig. 3B) and the number of action potentials evoked by 2 times and 3 times rheobase current stimulation of recorded neurons (Fig. 3C and D).

### CBS inhibitor AOAA reduces sodium current densities

**Figure 4:**
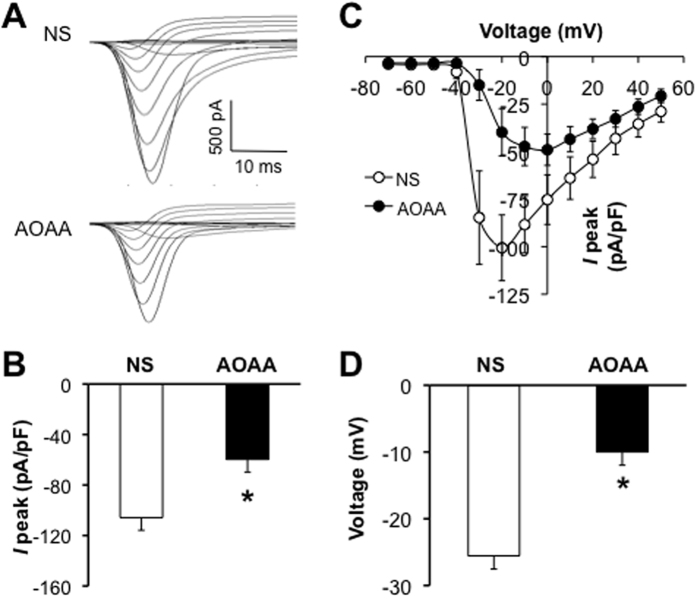
Inhibition of CBS by AOAA suppressed sodium current densities. (**A**) The total voltage-gated Na^+^ currents recorded from NS (top) and AOAA group (bottom). The membrane potential was held at −60 mV and voltage steps were from −70 mV to +50 mV with 10 mV increment and 80 ms in duration. (**B**) Bar graphs showed the mean peak current densities of sodium current from NS and AOAA group. The current density (in pA/pF) was calculated by dividing the current amplitude by cell membrane capacitance. The current density of DiI-labeled neurons from AOAA group was significantly decreased when compared with NS group (NS: −105.8 ± 18.1 pA/pF, n = 9; AOAA: −59.7 ± 9.8 pA/pF, n = 11, *p < 0.05). (**C**) Current vs. Voltage (I-V) curves were plotted from the all recorded cells. AOAA treatment did not alter the reversal potentials of total NaV currents. (**D**) Bar graphs showed that the membrane voltage, at which the current was maximally activated, was significantly depolarized in AOAA group when compared with NS group (NS, −25.6 ± 2.4 mV, n = 9; AOAA, −10 ± 4.9 mV, n = 11, *p < 0.05).

We next examined effects of AOAA on sodium currents of L_5_-L_6_ DRG neurons from LDH rats. In the NS-treated group, the sodium current density was −105.8 ± 18.1 pA/pF (n = 9 cells). In the AOAA-treated group, the sodium current density was −59.7 ± 9.8 pA/pF (n = 11 cells). AOAA injection remarkably suppressed sodium current density when compared with NS group (Fig. 4A and B, *p < 0.05). However, AOAA injection did not markedly change the reversal potentials. The average reversal potentials were +78.4 mV and +86.9 mV for NS- and AOAA-treated group, respectively (Fig. 4C). This indicated that ion permeability of DRG neurons was not significantly altered after AOAA injection. However, the average voltage where the maximal current achieved was greatly depolarized after AOAA injection when compared with NS group (Fig. 4D, *p < 0.05). The average voltage was −25.6 ± 2.4 mV (n = 9 cells) and −10 ± 4.9 mV (n = 11 cells) for NS and AOAA group, respectively.

### Treatment with AOAA rightshifts inactivation curve of sodium currents

**Figure 5:**
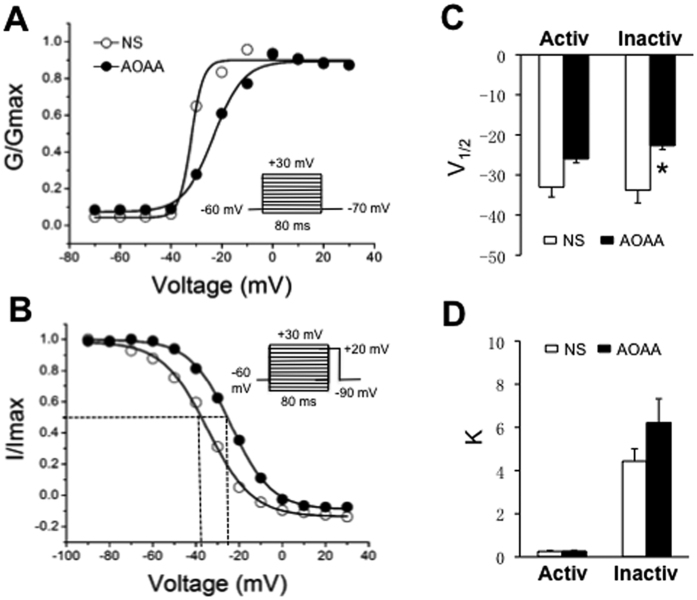
Effect of CBS inhibitor on activation and steady-state inactivation curves of sodium currents. (**A**) For activation curves, the sodium currents were generated by 2 voltage pulses in 10 mV increment steps from −70 to +30 mV in a DiI-labeled DRG neuron from a rat treated with NS and a rat treated with AOAA. The reversal membrane potential (Vrev) in this recording condition was +78 mV. At different test potentials, membrane conductance (G) was measured by dividing the peak sodium current by the current driving force (Vm-Vrev) and was normalized to that recorded at +30 mV (Gmax). Data were fitted with the modified Boltzmann equation: G/Gmax = 1/{1-exp[(V-V_1/2_)/k]}, where V is membrane potential, V_1/2_ (V half) is the membrane voltage at which the current was half-maximally activated, and k is the slope factor. AOAA treatment did not alter the activation curve compared with NS group. There were no significant differences of V_1/2_ (**C**) and k (**D**) between NS and AOAA group. (**B**) For steady-state inactivation curves, a conditional step of various voltages from −90 to +30 mV with 10 mV increment. These inactivation curves are representative curves of one neuron from a rat treated with NS and one neuron from a rat treated with AOAA, respectively. The peak current amplitude was normalized to that recorded at a −90 mV conditioning step (Imax). Data were plotted as a function of conditional step potentials and fitted with the negative Boltzmann equation: I/Imax = 1/{1-exp[(V_1/2_-V)/k]}. AOAA treatment induced the inactivation curve rightward shift compared with the control (NS). (**C**) Bar graphs showed that there was no difference of V_1/2_ of activation curve between NS and AOAA-treated rats. The V_1/2_ of activation curve was −26.0 ± 2.9 mV (n = 10) for neurons from AOAA rats and −33.0 ± 2.5 mV (n = 8) for neurons from NS rats (p > 0.05, two sample t-test); However, AOAA treatment significantly reduced the V_1/2_ of inactivation curves. The V_1/2_ of inactivation curve was −33.8 ± 3.2 mV (n = 9) for neurons from NS rats and −22.7 ± 3.9 mV (n = 7) for neurons from AOAA-treated rats (*p < 0.05, two sample t-test). (**D**) AOAA treatment did not significantly alter the k values of activation curves and inactivation curves.

Since AOAA injection significantly reduced the peak current density of total sodium currents, we next examined the effect of AOAA on voltage dependence of sodium currents of DRG neurons innervating the hindpaw. The conductance (G) and voltage (V) relationship curves were fitted with a modified Boltzmann equation to obtain the half-maximal activation potential (V_1/2_) and the slope factor (*k*). The G-V curve from NS-treated rats had a V_1/2_ of −33.0 ± 2.5 mV and *k* of 0.26 ± 0.05 (n = 8 cells; Fig. 5A,C and D). The G-V curve from AOAA-treated rats had a V_1/2_ of −26.0 ± 2.9 mV and *k* of 0.27 ± 0.04 (n = 10 cells). AOAA administration did not significantly change the steady-state activation curve (Fig. 5A,C and D, p > 0.05, two sample *t* test). We then examined roles of AOAA on the voltage dependence of steady-state inactivation of sodium channel currents of the DRG neurons (Fig. 5B,C and D). The *I*-V curve of steady-state inactivation from NS-treated rats had a V_1/2_ of −33.8 ± 3.2 mV and *k* of 4.43 ± 0.57 (n = 9 cells; Fig. 5B,C and D). In AOAA-treated group, the V_1/2_ and *k* of the *I*-V curve were −22.7 ± 3.9 mV (n = 7 cells) and 6.22 ± 1.11 (n = 7 cells), respectively. Statistical analysis showed that AOAA administration significantly altered the V_1/2_ of the inactivation curve of DRG neurons innervating the hindpaw (Fig. 5C, *p < 0.05).

### AOAA treatment suppresses expression of Na_V_1.7 and Na_V_1.8

**Figure 6:**
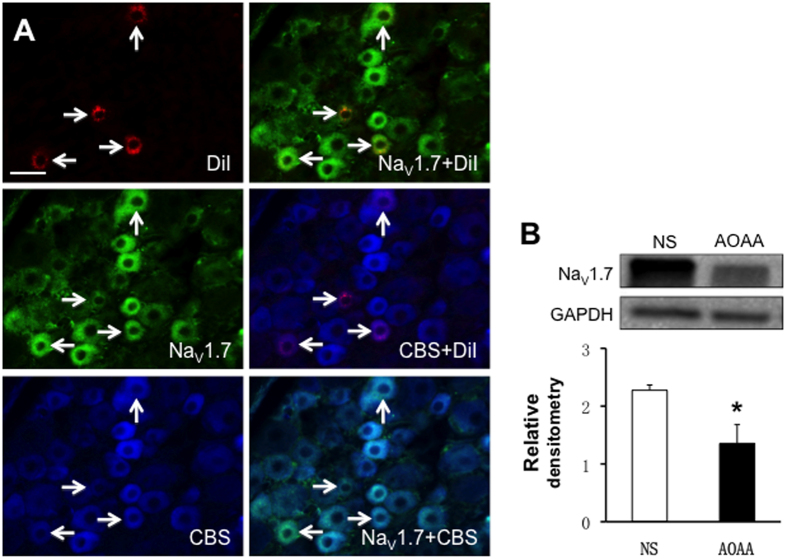
Antagonism of CBS inhibitor suppressed expression of Na_V_1.7. (**A**) Triple-labeling techniques showed that CBS (blue)- and Na_V_1.7 (green)-like immunoreactivities were colocalized in DiI (red) labeled DRG neurons innervating the hindpaw. Bar = 50 μm. (**B**) AOAA treatment significantly reduced expression of the Na_V_1.7 in L_5–6_ DRGs when compared with NS group (n = 4 rats for each group, *p < 0.05).

To further investigate the roles of CBS on voltage-gated sodium channels, we first determined whether CBS was co-expressed with Na_V_1.7 in L_5_-L_6_ DRG neurons innervating the hindpaw by triple-labeling techniques (Fig. 6). Immunohistochemistry experiments demonstrated that all L_5_-L_6_ DRG neurons that were immunoreactive for CBS also were positive for Na_V_1.7, and that all L_5_-L_6_ DRG neurons that were immunoreactive for Na_V_1.7 also were positive for CBS (Fig. 6A, arrows). Next, we investigated the effect of CBS treatment on Na_V_1.7 expression. AOAA was injected intrathecally (i.t., 10 μg/kg body weight) once per day for 7 consecutive days in LDH rats. Control rats received the equal volume of NS. The relative densitometry of Na_V_1.7 was 2.27 ± 0.18 (n = 4) and 1.36 ± 0.45 (n = 4) for NS and AOAA, respectively. AOAA injection markedly lowered the expression of Na_V_1.7 in L_5_-L_6_ DRGs of LDH rats (Fig. 6B, *p < 0.05).

**Figure 7:**
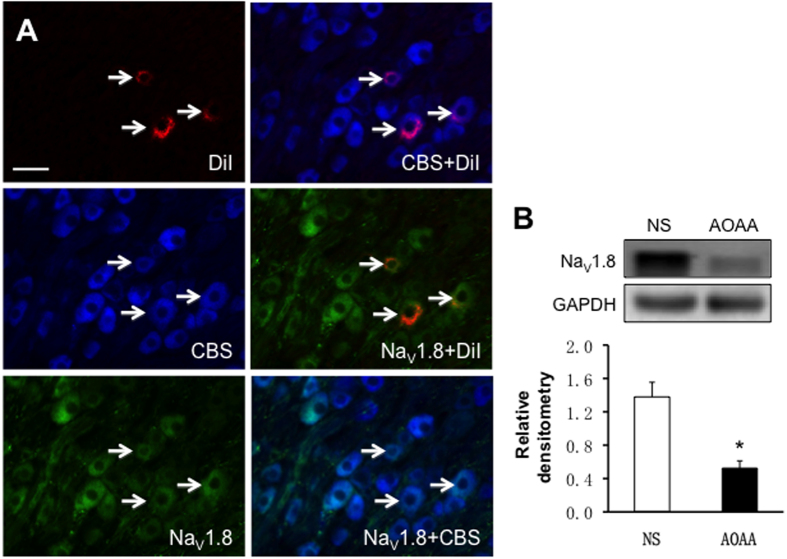
Antagonism of CBS inhibitor suppressed expression of Na_V_1.8. (**A**) Triple-labeling techniques showed that CBS (blue)- and Na_V_1.8 (green)-like immunoreactivities were colocalized in DiI (red) labeled DRG neurons innervating the hindpaw. Bar = 50 μm. (**B**) AOAA treatment significantly reduced expression of the Na_V_1.8 in L_5–6_ DRGs when compared with NS group (n = 4 rats for each group, *p < 0.05).

In addition, we investigated whether CBS was co-expressed with Na_V_1.8 in L_5_-L_6_ DRG neurons innervating the hindpaw by triple-labeling techniques (Fig. 7). Immunohistochemistry experiments demonstrated that all L_5_-L_6_ DRG neurons that were immunoreactive for CBS were also positive for Na_V_1.8, and that all L_5_-L_6_ DRG neurons that were immunoreactive for Na_V_1.8 were also positive for CBS (Fig. 7A, arrows). After intrathecal injection of CBS inhibitor AOAA or NS, expression of Na_V_1.8 in L5-6 DRGs was measured from LDH rats. The relative densitometry of Na_V_1.8 was 1.38 ± 0.35 (n = 4) and 0.52 ± 0.18 (n = 4) for NS and AOAA, respectively. AOAA administration dramatically reduced expression of Na_V_1.8 in L_5_-L_6_ DRGs of LDH rats (Fig. 7B, *p < 0.05).

**Figure 8:**
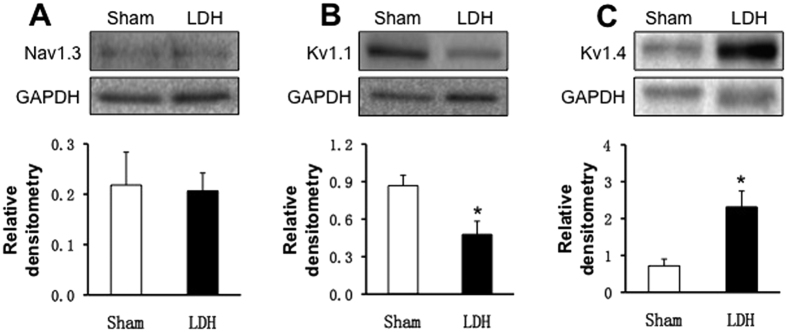
Expression of Na_V_1.3, K_V_1.1 and K_V_1.4. (**A**) There was no difference of Na_V_1.3 expression between LDH and Sham (n = 4 for each group). (**B**) The expression of the K_V_1.1 in L_5–6_ DRGs was significantly reduced in LDH rats when compared with Sham group (n = 4 rats for each group, *p < 0.05). (**C**) The expression of the K_V_1.4 in L_5–6_ DRGs was greatly increased in LDH group of rats when compared with Sham group (n = 3 rats for each group, *p < 0.05).

The expression of Na_V_1.3, K_V_1.1 and K_V_1.4 in L5-6 DRGs were also examined from sham and LDH rats. As shown in Fig. 8, there was no significant difference in Na_V_1.3 expression between LDH and Sham group of rats (Fig. 8A, n = 4 for each group). However, expression of K_V_1.1 was remarkably decreased in LDH group when compared with Sham (Fig. 8B, n = 4 for each group, *P < 0.05). In contrast, expression of K_V_1.4 was markedly upregulated in LDH group when compared with Sham (Fig. 8C, n = 3 for each group, *P < 0.05).

## Discussion

This study showed that administration of CBS inhibitor AOAA remarkably suppressed expression of Na_V_1.7 and Na_V_1.8 in DRGs of LDH rats. Inhibition of CBS activity also reversed neuronal hyperexcitability and reduced the sodium current densities of DRG neurons innervating the rat hindpaw. More importantly, inhibition of CBS significantly attenuated the PWL and PWT of LDH rats. Together with our previous report^4^, our findings suggest that endogenous H_2_S signaling pathway might play a critical role in regulating VGSC activities in an LDH rat model of pain hypersensitivity.

An important feature of the present study was the intrathecal injection of AOAA. The dose of AOAA used in this study was selected based on our previous *in vivo* experiments in a rat model of inflammatory pain and visceral hypersensitivity^4^,^12^,^15^. Since hydroxylamine, another CBS inhibitor, has showed an inhibitory effect on COX-1^16^, we used AOAA rather than hydroxylamine to avoid the unwanted effect. Because there was no significant effect of the same dose of AOAA observed in control rats in our previous study^15^, the present data suggest that the AOAA-produced antinociceptive effect was not a non-specific analgesic effect. In addition, we showed that *in vitro* incubation of AOAA did not produce any effect on the neuronal excitability, further indicating that the effect of *in vivo* application of AOAA was not a toxic effect or non-specific effect. Together with our previous studies^4^,^12^,^14^, CBS-H_2_S signaling pathway plays an important role in signaling pain hypersensitivity in various pathophysiological conditions.

The merging evidence has suggested that CBS inhibitors produced antinociceptive effects in animal models of inflammatory pain, but the detailed mechanisms underlying the antinociceptive effects of AOAA in LDH rats remain largely unknown. We showed in the present study that the significant reduction of sodium current densities was observed in DRG neurons innervating the hindpaw of LDH rats after chronic intrathecal injection of CBS inhibitor AOAA. In addition, CBS administration partially reversed the PWL and PWT of LDH rats. These data indicate an important role of CBS-H_2_S signaling in pain hypersensitivity of LDH. CBS is one of three major endogenous enzymes responsible for generation of H_2_S^17^,^18^. Once endogenously synthesized, H_2_S directly or indirectly modulate functions and expressions of various ion channels, such as voltage-gated sodium channels^7^,^12^ and voltage-gated potassium channels^19^. In the present study, we provided additional evidence to support the idea that CBS-H_2_S signaling regulated VGSC activities of DRG neurons innervating the rat hindpaw. Firstly, AOAA treatment suppressed Na_V_1.7 and Na_V_1.8 expression in L_5_-L_6_ DRGs of LDH rats. Secondly, AOAA administration also reduced the total sodium current density of DRG neurons innervating the hindpaw of LDH rats. The suppression of Na_V_1.7 and Na_V_1.8 expression would give rise to the reduction in total sodium channel currents of DRG neurons. Thirdly, AOAA treatment reversed the hyperexcitability of DRG neurons innervating the hindpaw of LDH rats. The reduced VGSC current densities would explain the reversal of neuronal excitability by application of AOAA. In addition, AOAA treatment also right shifted the inactivation curve. Together with our previous reports^9^, the present study provided additional evidence to show that CBS-H_2_S is involved in regulation of sodium channel function and expression. Since our previous report showed that NaHS, H_2_S donor, significantly potentiated both the TTX-sensitive and TTX-resistant sodium channel current density^14^, we hypothesized that CBS-H_2_S signaling pathway regulates VGSCs might be chronic pain specific. However, this needs to be further confirmed. Of note is that other subtypes of VGSCs might be also regulated by CBS-H_2_S signaling. In the present study, Na_V_1.3 subtypes may not be involved because the expression of Na_V_1.3 was not altered in this setting.

The detailed Mechanisms by which H_2_S regulates VGSCs remain largely unknown. One of likely mechanisms for sulfide signaling is persulfidation of target proteins since polysulfide functions as pronociceptive substance^20^. However, this is challenged by the relatively poor reactivity of H_2_S toward oxidized thiols such as disulfides, and the low steady-state concentration of H_2_S. Another signaling mechanism is sulfide oxidation pathways, which is considered to be primary mechanism for disposing of excess sulfide and to generate a series of reactive sulfur species, thus modifying the target proteins, such as VGSCs. Oxidative stress is a key contributor to neuronal cell dysfunction and associated pathogenesis of many tissues and systems. As an anti-oxidant gasotransmiter, H_2_S protects cells against oxidative stress^21^,^22^. The third possible mechanism, which needs to be further confirmed, is that H_2_S may directly phosphorylate sodium channels^14^. Since ion channels are emerging as an important family of target proteins for modulation by H_2_S^4^,^23^, and both VGSCs and H_2_S are involved in cellular responses to inflammation and tissue injuries, the detailed mechanisms for sensitization of VGSCs by H_2_S need to be determined ultimately. Of note is that recent studies showed that sodium channels are not the sole target of H_2_S-induced pain hypersensitivity. T-type calcium channels^23^,^24^, TRPV1^25^,^26^, TRPA1^20^ and NMDA receptors^27^ have been indicated to play a crucial role in nociception. Conversely, H_2_S has been reported to mediate an anti-nociceptive effect by opening K(ATP) channels^28^,^29^ and to prevent the development of opioid withdrawal-induced hyperalgesia by suppressing spinal calcitonin gene-related peptide expression^30^. In the present study, since the expression of K_V_1.1 and K_V_1.4 went to opposite directions, it is difficult to determine the roles of voltage-gated potassium channels under LDH conditions. Therefore, It is worthy of further investigation.

In summary, the present studies demonstrated that inhibition of the endogenous H_2_S producing enzyme CBS significantly reduced expression of Na_V_1.7 and Na_V_1.8 and suppressed the total voltage-gated sodium channel currents of the DRG neurons from LDH rats. Since inhibition of CBS reverses hyperexcitability of primary sensory neurons and attenuates colonic pain hypersensitivities, our data further indicate that targeting the CBS-H_2_S pathway might be a therapeutic strategy for chronic pain relief.

## Additional Information

**How to cite this article**: Yan, J. *et al*. Inhibition of cystathionine *β*-synthetase suppresses sodium channel activities of dorsal root ganglion neurons of rats with lumbar disc herniation. *Sci. Rep.* **6**, 38188; doi: 10.1038/srep38188 (2016).

**Publisher's note:** Springer Nature remains neutral with regard to jurisdictional claims in published maps and institutional affiliations.
